# Association between problem gambling and personality traits: a longitudinal study among the general Norwegian population

**DOI:** 10.3389/fpsyg.2023.1241365

**Published:** 2023-11-29

**Authors:** Puneet Kaur, Tony Leino, Razieh Chegeni, Eilin K. Erevik, Rune A. Mentzoni, Ståle Pallesen

**Affiliations:** ^1^Department of Psychosocial Science, University of Bergen, Bergen, Norway; ^2^Optentia Research Focus Area, North-West University, Vanderbijlpark, South Africa; ^3^Norwegian Competence Center for Gambling and Gaming Research, University of Bergen, Bergen, Norway; ^4^Department of Health Promotion, Norwegian Institute of Public Health, Bergen, Norway; ^5^Department of Psychology, PROMENTA Research Center, University of Oslo, Oslo, Norway

**Keywords:** problem gambling, personality traits, cross-lagged, longitudinal study, quantitative study

## Abstract

**Objective:**

The present study investigates the longitudinal relationship between problematic gambling (PG) and the five factor model’s personality traits using autoregressive cross-lagged models.

**Methods:**

The data used in the current study was collected by a national survey in 2013 (*n* = 10,081) and a follow-up study (*n* = 5,848) in 2015. PG was measured using Canadian Problem Gambling Index (CPGI) while personality was assessed using Mini-International Personality Item Pool (MINI-IPIP). Participants who completed the CPGI and all the personality items during both waves (*n* = 2,702) were analysed.

**Results:**

The results show that neuroticism had positive cross-lagged associations with CPGI. In contrast, conscientiousness and agreeableness in 2013 were found to have inverse cross-lagged effect on CPGI in 2015. Finally, openness and extraversion did not have any cross-lagged associations with CPGI.

**Conclusion:**

PG poses serious negative implications for the involved individuals as well as their associated close social circle. Hence, it is important to understand predictors of PG for prevention purposes. Personality traits are one of the influential frameworks for examining uncontrolled psychopathological behaviors like PG. The study findings offer significant theoretical as well as practical implications.

## 1 Introduction

Occasional gambling is often experienced as an enjoyable experience (Molde et al., 2019). However, scholars caution that the gratifying nature of gambling may lead to excessive or incontrollable gambling for some, often denoted problematic gambling (PG) (Graves, 2018). Gambling problems can be defined as maladaptive gambling behavior that disrupts personal, family and/or vocational pursuits (Hodgins and El-Guebaly, 2000). In line with the gratifying nature of gambling, the literature suggests that some individuals gamble as a strategy for mood modification and temporary escape from the negative states (Ciccarelli M. C. et al., 2016).

Even though the percentage of the general population which is deemed as problematic gambler is relatively low, it is still considered as a major health concern owing to the social, psychological and financial implications associated with such gambling (Delfabbro and King, 2020). The implications of gambling can be considered both at the individual and societal level. At the individual level, problem gamblers can suffer cognitively [e.g., cognitive distortion, poor decision making (Ciccarelli M. C. et al., 2016); foreshortened time horizon (Ciccarelli M. et al., 2016)], affectively [e.g., mood disorder (Lorains et al., 2011)], and behaviourally [e.g., substance use disorder, anti-social personality disorder (Dowd et al., 2019)]. Furthermore, problem gamblers are often found to experience various mental health issues such as emotional distress, anxiety, depression, and impulsivity among others (Ciccarelli M. C. et al., 2016). Finally, scholars also suggest that PG poses various financial challenges posed due to bankruptcy, debts and increased health-care costs (Graves, 2018). From the societal perspective, PG is associated with disturbed family life, strained relationships with family, friends and colleagues (Onyedire et al., 2019) and impairment of daily functioning (Madey, 2019). Kalischuk et al. (2006) report that a problematic gambler could affect about five to fifteen other people.

Scholars have suggested that personality traits may be influential in explaining and understanding different forms of psychopathological behaviors including PG (Strømme et al., 2021). Personality is regarded as a psychophysical structure within individuals which is dynamic in nature and affect the way humans respond to their environment (Allport, 1937). The empirical evidence of significant associations between personality traits and PG further strengthens the inference that personality can play an influential role in the development of PG (Dowd et al., 2019). One of the most influential contemporary trait theories is the five-factor model of personality. This model differentiates between five main personality dimensions: (1) *Neuroticism* (e.g., being nervous and anxiety prone), (2) *Extraversion* (e.g., being talkative and outgoing), (3) *Openness to experience* (e.g., being imaginative and intellectually oriented), (4) *Agreeableness* (e.g., being sympathetic and warm), and (5) *Conscientiousness* (e.g., being organized and prompt) (Wiggins, 1996). According to evolutionary perspectives the five dimensions are closely linked to solving adaptive problems, e.g., in terms of deciding who will be a burden, who will be a good cooperator and who will work industriously (Buss, 1991). Scholars have examined the association between the dimensions of the five factor model of personality and PG [e.g., (Brunborg et al., 2016; Reardon et al., 2019)].

The extant research on the topic reports that the typical profile of the adult problematic gambler includes being high on neuroticism and low on conscientiousness and agreeableness (Tackett et al., 2015; Brunborg et al., 2016). Other studies suggests that problematic gamblers are found to be low in openness (Madey, 2019). Similarly, adolescents and young adults low in conscientiousness and agreeableness and high on neuroticism represent a vulnerable group, more likely to develop PG compared to their counterparts (Tackett et al., 2015; Reardon et al., 2019).

Although some associations between the five-factor model of personality and PG has been established our review of prior literature suggest three research gaps: (a) there is mixed findings regarding the possible associations due to differences in the demographic profile of respondents, culture, choice of measurement and research design. For example, scholors have suggested absence of significant association between neuroticism and PG [e.g., (Hwang et al., 2012)] while some studies suggest otherwise (Tackett et al., 2015; Pallesen et al., 2016); (b) Most prior studies have relied on small sample sizes (Tackett et al., 2015; Mackinnon et al., 2016; Reardon et al., 2019) with some exceptions (Brunborg et al., 2016). Furthermore, prior literature is predominately based on cross-sectional studies (Brunborg et al., 2016; Reardon et al., 2019) with an exception of few longitudinal studies (Mackinnon et al., 2016); (c) To the best of our knowledge, the prior literature lacks knowledge regarding the nature of reciprocal association among different personality traits and PG. The current study aims to fill the aforementioned gaps by adding new knowledge regarding the reciprocal association of personality traits and PG through a two-year long longitudinal panel study, based on a representative sample of 2,702 Norwegian gamblers. The initial hypotheses for the study are: (i) different personality traits in 2013 influence CPGI in 2015 and (ii) CPGI in 2013 influences different personality traits in 2015.

## 2 Materials and methods

### 2.1 Participants

The present study takes into consideration all the participants who completed the CPGI and all the personality items during both waves. The age range for the analytic sample varied from 16 to 74 years with mean age of 50.66 years (SD = 13.24 years) and consists of 49.5% females (*n* = 1,338) and 50.5% males (*n* = 1,364) in 2013. Table 1 presents details of the demographic variables of the study participants.

**Table 1:** Descriptive statistics of the study participants in 2013.

Demographic		
**Age**		
Mean (SD)	50.4 (13.3)	
**Gender**	*N*	%
Male	1,364	50.5%
Female	1,338	49.5%
**Civil status**		
Married/Cohabiting	2052	76.5%
Single/Separated/Divorced/Widowed	631	23.5%
**Education**		
Primary school or less	178	8,6%
High or vocational school	569	27,6%
College/university (1–4 years)	883	42,8%
College/university (5 years or more)	432	21,0%
**Income**		
0–499,999 NOK	1815	67,8%
500,000–999,999 NOK	779	29,1%
1 Mill NOK or more	83	3,1%

Average exchange rate for 1€ in 2013 was 7.81 NOK.

### 2.2 Instruments

#### 2.2.1 Gambling participation

The questionnaire contained a definition of gambling whereupon the respondents were asked if they had participated in gambling during the last 12 months (yes/no). Gambling was defined as “*a game where money is bet on a specific outcome of an event or draw and where you can win cash prizes (*e.g.*, Lotto, Tipping, scratch cards, casino games* etc.*).”* Those who endorsed were asked to complete the Canadian Problem Gambling Index.

#### 2.2.2 Canadian problem gambling index

The CPGI scale measured PG behavior and its probable consequences using nine items with response alternatives ranging from “never” (0) to “always” (Hodgins and El-Guebaly, 2000; Ferris and Wynne, 2001). The scale has a total of nine items were five items are examining the PG behavior while the other four items are investigating its probable consequences. The composite score ranges from 0 to 27 enabling grouping of the participants into four different severity levels of problematic gambling. The four severity levels reflect non-problem gambler (score – 0), low risk gambler (score 1–2), moderate risk gambler (score 3–7), and problem gambler (score 8–27). In the present analysis the composite score was used by adding the score of each item for each individual participant where higher score reflects more gambling problems. In 2013 the CPGI scale had a Cronbach’s alpha of 0.87 while it was 0.81 in 2015.

#### 2.2.3 Mini-international personality item pool

The MINI-IPIP scale was used for assessing the personality of the respondents (Donnnellan et al., 2006). The scale consists of 20 items measuring the traits of the five-factor model of personality namely: extraversion, agreeableness, conscientiousness, neuroticism, and openness. Each personality trait was measured using four items, with response alternatives ranging from “very wrong” (Molde et al., 2019) to “very correct” (Delfabbro and King, 2020). The Cronbach’s alpha values in wave 1 were as follows: 0.78 for extraversion, 0.69 for agreeableness, 0.65 for conscientiousness, 0.66 for neuroticism and 0.68 for openness. For wave 2, the corresponding values were 0.77, 0.70, 0.64, 0.66 and, 0.68, respectively.

### 2.3 Procedure

The longitudinal study was conducted among the general adult Norwegian population on the behalf of the Norwegian Gaming Authority, over a period of two years. The study was approved by Regional Committee for Medical and Health Related Ethics in Western Norway (REK-Vest, project no. 2013/120). The first wave survey was conducted in 2013 where 24,000 people were invited to participate. In total 10,081 valid responses were received, generating a response rate of 42% after removal of those who could not be reached due to wrong addresses. The follow-up wave was conducted in 2015 where the participants of the 2013 study were invited. Of the 10,081 who responded in the 2013 wave 9,741 respondents were reachable, of which 5,809 responded, amounting to a response rate of 59.6%. In both waves, a maximum of two reminders were sent to those who did not respond. Furthermore, all respondents were informed about their participation in a lottery where they had the chance to win the gift voucher of NOK 500 upon answering. The Norwegian Gambling Authority and the Regional Committee for Medical Research and Health Research Ethics in Western Norway had no objections with regards to the usage of lottery as a mode for increasing the response rate. Furthermore, the procedure did not reflect gambling in itself since it did not involve staking money or any other materials of value. Moreover, prior literature reports that incentives could increase response rates together with representativeness of the sample (Olsen et al., 2012). The participants reporting problems through the survey were not contacted actively by the researchers. However, contact information of the researchers was provided to the participants (phone and emails) and the researchers could upon being contacted refer to the ones needing help with relevant treatment facilities.

### 2.4 Statistical analysis

As mentioned before, respondents who completed both CPGI and all personality traits in both waves were considered for the analysis. Furthermore, the participants with no missing value on any composite personality scores were included. Thus, resulting in the effective sample size of 2,702 respondents.

**Figure 1:**
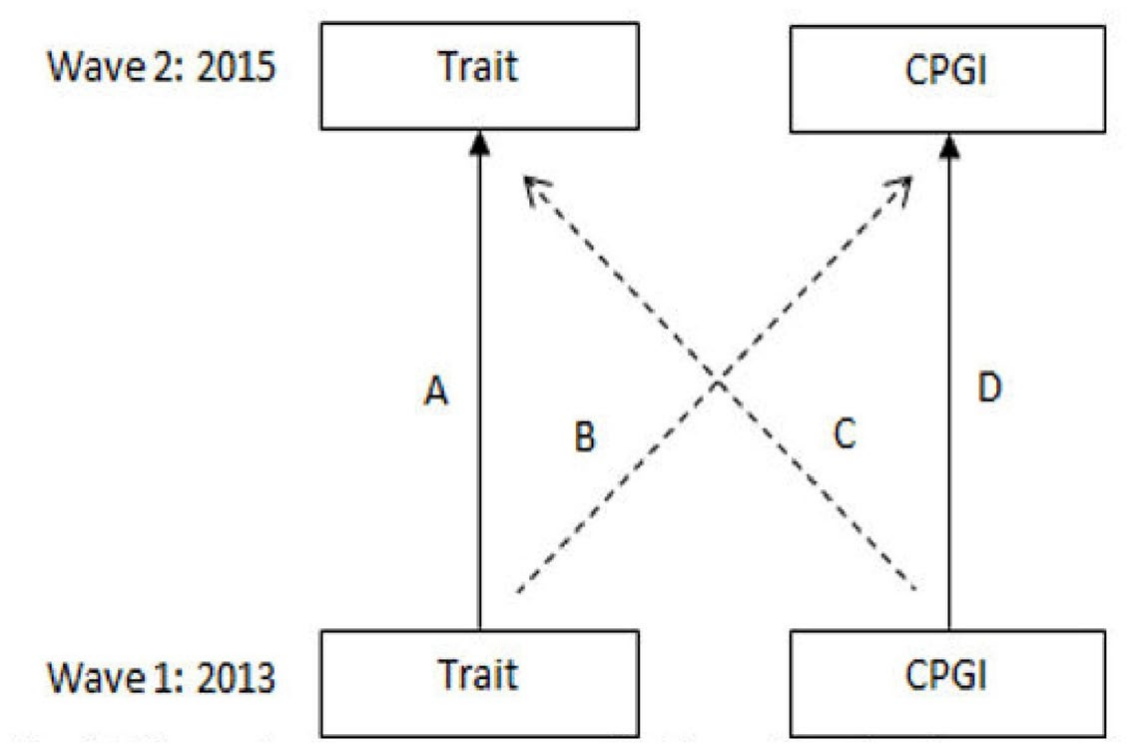
Research Model.

Analyses were conducted in R (version 4.1.1) using the lavaan package (0.6–9) (R Core Team, 2021). An autoregressive cross lagged model with observed indicators was tested to measure the cross-lagged associations among PG and different personality traits (see Figure 1). Autoregressive cross-lagged models examine the longitudinal associations between two constructs over a period of time while controlling for the stability of each construct over time. Moreover, the possibility for the estimation of reciprocal effects on change between two variables over time, together with maintaining of the temporal order offers an advantage compared to other longitudinal modelling approaches (Selig and Little, 2012). For both the CPGI and the five personality traits composite scores were calculated for each wave, respectively. The composite score for all the items on each specific personality trait and CPGI was calculated for first and second time point. The analysis used robust maximum likelihood estimation with Satora-Bentler scaled chi-squared test involving robust standard errors. As a 2-wave autoregressive model are just-identified (contains no degrees of freedom) traditional model fit SEM statistics are not available. Hence, the analysis was conducted by running four different models with criterion based fit indices *Akaike’s information criteria* (AIC), *Bayesian information criteria* (BIC) and *Expected cross-validation index* (ECVI) in addition to traditional model fit indices of *Model Chi-Square* (χ^2^), *Comparative fit index* (CFI), *Tucker Lewis index* (TLI), *Root mean square error of approximation* (RMSEA), *Standardized root mean square residual* (SRMR) and *95% confidence intervals* (95% CI) were available. The null-model (M_0_) represents a model with no path effects. The stability model (M_1_) represents a model where only the auto-regressive paths are investigated. The cross-lagged model (M_2_) represents a model where only the cross-lagged effects are investigated. Finally, the just-identified model (M_3_) represents a model where both cross-lagged and autoregressive paths are investigated. The idea behind just-identified model to evaluate the proposed paths rather than how well the data fits the model. However, to compare the just-identified model to the other models (M_0_, M_1,_ and M_2_) criterion based fit indices are used where lower values for AIC, BIC and ECVI indicated a better and more pragmatic fitted model.

## 3 Results

The descriptive statistics and correlations among the study constructs over the time of two years are reported in Table 2. Overall, Table 2 show that there is no association between extraversion and openness and CPGI. The association between agreeableness, neuroticism, and consciousness ranges from marginal to small. Furthermore, there is a strong within correlation (WC) between all personally traits in 2013 and 2015, and a medium association between CPGI in 2013 and 2015. Table 3 presents four models tested as a part of the analysis containing details of their model fit and path estimates. Among the four tested models, cross-lagged associations were only tested in cross-lagged models and just-identified models. In general, the null model and cross-lagged models were found to have poor model fit for all the personality traits, mainly due to high AIC, BIC and ECVI values compared to the stability and just-identified model for all the personality traits. In contrast, the stability model and just-identified model were found to have good model fit for the different personality factors. However, the just-identified model was preferred over the stability model despite being a better model in the case of openness and extraversion, because the stability model only tested autoregressive paths.

**Table 2:** Descriptive statistics and correlations between personality traits and CPGI in 2013 and 2015.

Variables	Mean	SD	SKW	Correlations		
				CPGI 2013	CPGI 2015	WC
Extraversion 2013	13.85	3.39	−0.31	−0.02^NS^	−0.03^NS^	–
Extraversion 2015	13.68	3.38	−0.29	−0.03^NS^	−0.05^NS^	0.79
Agreeableness 2013	16.66	2.63	−0.85	−0.08	−0.12	–
Agreeableness 2015	16.63	2.68	−0.89	−0.06	−0.12	0.68
Conscientiousness 2013	16.18	2.79	−0.59	−0.12	−0.11	–
Conscientiousness 2015	16.21	2.77	−0.63	−0.09	−0.12	0.70
Neuroticism 2013	9.79	3.27	0.23	0.09	0.09	–
Neuroticism 2015	9.88	3.25	0.25	0.11	0.10	0.67
Openness 2013	13.59	3.27	−0.09	0.01^NS^	0.02^NS^	–
Openness 2015	13.57	3.29	−0.09	0.00^NS^	−0.01^NS^	0.70
CPGI 2013	0.30	1.34	10.65	–	–	–
CPGI 2015	0.28	1.09	9.60	–	–	0.43

*N* = 2,702. CPGI = Canadian problem gambling index. Extraversion = Sum score of Mini-IPIP items 1, 6R, 11, 16R. Agreeableness = Sum score of Mini-IPIP items 2, 7R, 12, 17R. Conscientiousness = Sum score of MINI-IPIP items 3, 8R, 13, 18R. Neuroticism = Sum score of Mini-IPIP items 4, 9R, 14, 19R. Openness = Sum score of Mini-IPIP items 5, 10R, 15R, 20R, WC = Within correlation, R = Reversed, SKW = Skewness. All Correlations significant at *p* < 0.001 except NS = Not significant.

**Table 3:** A cross-lagged path model of personality and problem gambling.

	Standardized beta				Model fit									
	Trait→Trait Path A	Trait→CPGI Path B	CPGI→Trait Path C	CPGI→CPGI Path D	df	*χ^2^*	CFI	TIL	RMSEA	[CI 95%]	SRMR	AIC	BIC	ECVI
Extraversion														
M_0_: null model	–	–	–	–	4	170.98	0.001	−0.498	0.536	[0.469, 0.606]	0.285	45,881.765	45,917.176	1.181
M_1_: stability model	0.788**	–	–	0.432**	2	3.056^NS^	1.000	0.999	0.013	[0.000, 0.041]	0.011	42,708.460	42,755.674	0.007
M_2_: cross-lagged model	–	−0.027^NS^	−0.007^NS^	–	2	96.562	0.000	−2.003	0.759	[0.634, 0.892]	0.285	45,885.248	45,932.462	1.183
M_3_: just-identified model	0.788**	−0.025^NS^	−0.010^NS^	0.432**	0	0.000	1.000	1.000	0.000	[0.000, 0.000]	0.000	42,709.735	42,768.753	0.007
Agreeableness														
M_0_: null model	–	–	–	–	4	122.859	0.025	−0.462	0.448	[0.381, 0.517]	0.259	43,203.912	43,239.323	0.832
M_1_: stability model	0.679**	–	–	0.433**	2	19.792	0.990	0.971	0.062	[0.039, 0.089]	0.032	40,994.031	41,041.245	0.015
M_2_: cross-lagged model	–	−0.089^NS^	−0.042^NS^	–	2	64.827	0.028	−1.917	0.632	[0.505, 0.769]	0.254	43,198.300	43,245.513	0.830
M_3_: just-identified model	0.681**	−0.084**	−0.004^NS^	0.426**	0	0.000	1.000	1.000	0.000	[0.000, 0.000]	0.000	40,974.552	41,033.569	0.007
Conscientiousness														
M_0_: null model	–	–	–	–	4	133.049	0.031	−0.454	0.464	[0.398, 0.533]	0.265	43,688.411	43,723.821	0.891
M_1_: stability model	0.703**	–	–	0.433**	2	14.944	0.996	0.987	0.044	[0.025, 0.066]	0.023	41,308.290	41,355.499	0.010
M_2_: cross-lagged model	–	−0.105**	−0.085^*^	–	2	70.117	0.036	−1.892	0.654	[0.528, 0.790]	0.259	43,675.551	43,722.765	0.887
M_3_: just-identified model	0.704**	−0.060^*^	0.005^NS^	0.426**	0	0.000	1.000	1.000	0.000	[0.000, 0.000]	0.000	41,300.212	41,359.230	0.007
Neuroticism														
M_0_: null model	–	–	–	–	4	121.394	0.020	−0.470	0.442	[0.376, 0.511]	0.257	45,441.913	45,477.324	0.811
M_1_: stability model	0.671**	–	–	0.431**	2	25.651	0.990	0.970	0.063	[0.043, 0.086]	0.030	43,289.895	43,337.109	0.015
M_2_: cross-lagged model	–	0.102^*^	0.120**	–	2	65.542	0.032	−1.904	0.621	[0.494, 0.757]	0.251	45,417.129	45,464.343	0.802
M_3_: just-identified model	0.667**	0.052^*^	0.054**	0.428**	0	0.000	1.000	1.000	0.000	[0.000, 0.000]	0.000	43,270.344	43,329.362	0.007
Openness														
M_0_: null model	–	–	–	–	4	135.195	0.000	−0.501	0.466	[0.401, 0.535]	0.262	45,545.770	45,581.180	0.901
M_1_: stability model	0.705**	–	–	0.433**	2	1.958	1.000	1.000	0.000	[0.000, 0.032]	0.007	43,129.962	43,177.176	0.006
M_2_: cross-lagged model	–	0.079^*^	0.035^NS^	–	2	69.403	0.002	−1.995	0.659	[0.531, 0.796]	0.263	45,543.000	45,590.218	0.900
M_3_: just-identified model	0.705**	0.017^NS^	−0.009^NS^	0.433**	0	0.000	1.000	1.000	0.000	[0.000, 0.000]	0.000	43,132.532	43,191.550	0.007

*N* = 2,702. CPGI = Canadian problem gambling index. Path significance: ** *p* < 0.01, * *p* < 0.05 & NS = Not significant. CI = confidence interval. BIC=Bayesian information criterion. AIC = Akaikes information criterion. ECVI = Expected cross validation index.

For all personality traits, the final analysis (M3) showed significant trait to trait and CPGI to CPGI relationships (Path A and Path D) (see Table 3). For extraversion and openness, the cross-lagged association between trait to CPGI and CPGI to trait was not significant (Path B and Path C, respectively). For agreeableness and conscientiousness, there was a significant negative association between the trait in 2013 and CPGI score in 2015 (Paths B), *b* = −0.084, *p* < 0.01 and *b* = −0.060, *p* < 0.05, respectively, showing that greater agreeableness and conscientiousness in 2013 is associated with lower CPGI score in 2015. For neuroticism, there was a significant positive cross-lagged association between neuroticism in 2013 and CPGI in 2015 (Path B), *b* = 0.052, *p* < 0.05 and CPGI in 2013 and neuroticism in 2015 (Path C), *b* = 0.054, *p* < 0.01, suggesting that greater neuroticism is associated with higher CPGI score in 2015, and that higher CPGI score in 2013 is associated with greater neuroticism in 2015.

## 4 Discussion

The study findings report that neuroticism had significant positive cross-lagged effects with PG as neuroticism in 2013 was found to be positively correlated with CPGI in 2015, while CPGI in 2013 is found to influence neuroticism in 2015. An individual with higher levels of neuroticism is usually found to have higher likelihood of experiencing stress and anxiety and is emotionally vulnerable. Such individuals might engage in gambling as way for relieving their stress, which is in line with the escape hypothesis (Rogier et al., 2019). In line with the escape hypothesis, gambling is used as a strategy to escape negative or uncomfortable emotional states (Ciccarelli M. C. et al., 2016; Rogier et al., 2019). For example, gambling may be sought in order to escape from unpleasant feelings and states such as boredom and anxiety (Wulfert et al., 2005). Accordingly, gambling has been found to provide gratifying and enjoying experiences that could assist in temporary stress and anxiety management (Konietzny et al., 2007). The current finding regarding neuroticism and CPGI is in line with previous findings both on PG and other types of addictions (Whiting et al., 2019). Moreover, the prior literature has reported that PG creates stress and anxiety among the gamblers (Ste-Marie et al., 2006). Since individuals high on neuroticism have lower levels of self-control and are more prone to react strongly to stressful life events their PG condition may over time increase their neurotic tendencies, due to the negative consequences of PG (Paterson et al., 2020). Hence, the existence of a bidirectional cross-lagged influence between CPGI and neuroticism seems reasonable.

The personality trait of conscientiousness and agreeableness in 2013 was found to exert inverse cross-lagged influence on CPGI in 2015. The findings stand supported by prior literature showing that problematic gamblers have lower scores on conscientiousness (Brunborg et al., 2016; Whiting et al., 2019) and agreeableness (Tackett et al., 2015; Brunborg et al., 2016). The present findings indicate conscientiousness and agreeableness act as protective traits against developing PG. However, it should be noted that other factors (e.g., gender, age, education, attachment, and self-regulation) could mediate or moderate the association of conscientiousness and agreeableness with PG. It could be assumed that people with high scores on agreeableness avoid excessive gambling, as this typically causes interpersonal conflicts, which such individuals are motivated to avoid. People high on conscientiousness are characterized by high planning ability and self-control, which assumingly will put them in less risk of excessive gambling (Andreassen et al., 2013). In contrast, CPGI in 2013 had no cross-lagged influence on conscientiousness and agreeableness in 2015. Overall, the findings suggest that over time personality seems to exert stronger influence on CPGI than vice versa. This seems conceivable as personality is assumed to be relatively stable (Roberts et al., 2006) whereas CPGI seems more changeable (Nelson et al., 2009). These assumptions were also supported by the current data, showing stronger temporal stability for the personality traits (path A) than for PG (path D).

Extraversion and openness were found to share no cross-lagged correlation with CPGI. The absence of association of extraversion with PG is consistent with the prior literature (Brunborg et al., 2016). This reflects that extraversion as a personality trait seems not to influence PG even in the case of a longitudinal research setting. Similarly, openness did not influence CPGI over time. This finding contradicts the extant literature stating problem gamblers to be low on openness (Brunborg et al., 2016; Madey, 2019). In contrast, lack of association of CPGI in 2013 with both extraversion and openness in 2015 could be attributed to the conception that personality is relatively stable (Roberts et al., 2006).

It should be noted that personality traits associated with development of gambling problems are associated with other mental health problems. For example, several mental health and psychiatric conditions, such as depression and bipolar disorder are linked to the development of gambling problems (Kessler et al., 2008). Consequently, the relationship between personality and later gambling problems is possibly confounded by mental health problems or other psychiatric conditions.

### 4.1 Study implications

The present study offers different theoretical as well as practical implications. As pointed earlier, the majority of the extant literature provides information on the association between personality and CPGI at one point of time. However, the need for longitudinal investigations has also been emphasised in prior literature (Pallesen et al., 2016). As such the present study offers new knowledge on the temporal association of personality traits and PG. Practically, the findings could help practitioners and therapists in devising targeted prevention efforts for assisting individuals suffering from gambling disorder by focusing specifically on their personality traits. The study results suggest that neurotic individuals have higher tendency to develop PG. Similarly, problematic gamblers have tendency to further increase the level of stress and anxiety usually suffered by the neurotic individuals over the time. Hence, practitioners should focus on devising strategies for managing stress and anxiety of the problematic gamblers that would in turn aid in reducing PG. Consistent with prior literature, the individuals likely to develop PG have different personality characteristics compared to the general population (Tackett et al., 2015; Brunborg et al., 2016; Madey, 2019; Reardon et al., 2019). Overall, the study informs clinical psychologists that personality of an individual seems to have an influential role in determining their tendency to develop PG over time rather than the other way around. Hence, it is important clinical psychologists should consider performing personality screening and identifying individuals that are higher on neuroticism and low on conscientiousness and agreeableness. *For individuals identified with a personality associated with higher risk of PG, clinicians may consider psychoeducative steps to inform about how an individual’s personality puts them at risk for problematic gambling behaviour in the future*. Personality may also be relevant for the treatment process. Studies have for example shown that low scores on extraversion is associated with increased risk of drop-out from group treatment (MacNair and Corazzini, 1994) and that low scores on conscientiousness is associated with treatment non-compliance (Scherphof et al., 2014).

### 4.2 Limitations and strengths

The current study suffers from some limitations that should be noted. First, the study is based on self-reported data that could possibly introduce social desirability bias. Second, the study findings cannot be generalized with reservations, due to the national and research context. The study offers insights on gamblers from the general population using specific scales for measuring gambling problems As pointed by Brunborg et al. (2016), PG behavior could vary based on the chosen assessment strategy, geographical location, and cultural background of the respondents. The low ECVI value for the research model indicates that the obtained results have good predictability. Still, it would be useful to verify the findings of the present research in other settings. For example, future studies on this topic should use different scales for measuring PG and should include samples from other geographical locations. The generalization of the results might be influenced by attrition and selection. Still, the response rate is good as 59.6% of the original sample in 2013 also responded in 2015 (Guo et al., 2016). The findings also show that attrition is less likely to be problematic when examining relationships as compared to examining univariate distributions (Rindfuss et al., 2015). Furthermore, the current study reports the results across the full range of scores on CPGI, ranging from non-problem as well as problem gamblers (varying intensities of risk). As previously reported, the majority of the respondents of the present study were non-problem gamblers (86.12% in 2013 and 86.08% in 2015). This might influence the retrieved cross-lagged associations among different personality traits and PG. It should be noted that analysis has been conducted using robust model as an attempt to overcome such issues.

## 5 Conclusion

The present study adds to the extant knowledge on the nature of association between PG and personality. Through the lens of longitudinal research, the study sheds light on the temporal association among CPGI and personality traits. The proposed study hypotheses are partially supported. The results show that some personality traits (agreeableness, conscientiousness, and neuroticism) influence CPGI over time while only neuroticism has influence on CPGI over time. The study offers insightful information that could add depth in assisting individuals suffering from PG with focus on their personality.

## Data availability statement

The raw data supporting the conclusions of this article will be made available by the authors, without undue reservation.

## Author contributions

SP, RM, EE, and TL designed the study and collected the data. PK drafted the first version of the manuscript and did the revision that was approved by all the authors. TL conducted the analysis. SP, RM, EE, TL, and RC critically revised the manuscript. All authors contributed to the article and approved the submitted version.
